# Target Discovery of Matrine against PRRSV in Marc-145 Cells via Activity-Based Protein Profiling

**DOI:** 10.3390/ijms241411526

**Published:** 2023-07-16

**Authors:** Xiaoya Ling, Zhigang Cao, Panpan Sun, Hua Zhang, Yaogui Sun, Jia Zhong, Wei Yin, Kuohai Fan, Xiaozhong Zheng, Hongquan Li, Na Sun

**Affiliations:** 1 Shanxi Key Laboratory for Modernization of TCVM, College of Veterinary Medicine, Shanxi Agricultural University, Jinzhong 030600, China; 18735430346@163.com (X.L.);; 2 Laboratory Animal Center, Shanxi Agricultural University, Jinzhong 030600, China; 3 Centre for Inflammation Research, Queen’s Medical Research Institute, The University of Edinburgh, Edinburgh EH16 4TJ, UK

**Keywords:** matrine, PRRSV, ABPP, drug targets, probe

## Abstract

Porcine reproductive and respiratory syndrome (PRRS) seriously endangers the sustainable development of the pig industry. Our previous studies have shown that matrine can resist porcine reproductive and respiratory syndrome virus (PRRSV) infection. This study aimed to explore the anti-PRRSV targets of matrine in Marc-145 cells. Biotin-labeled matrine 1 and 2 were used as probes. MTT assay was used to determine the maximum non-cytotoxic concentration (MNTC) of each probe in Marc-145 cells. The anti-PRRSV activity of each probe was evaluated via MTT, qPCR and Western blot, and its anti-inflammatory activity was evaluated via qPCR and Western blot. The targets of matrine in Marc-145 cells were searched using activity-based protein profiling (ABPP), and compared with the targets predicted via network pharmacology for screening the potential targets of matrine against PRRSV. The protein–protein interaction networks (PPI) of potential targets were constructed using a network database and GO/KEGG enrichment analysis was performed. ACAT1, ALB, HMOX1, HSPA8, HSP90AB1, PARP1 and STAT1 were identified as potential targets of matrine, and their functions were related to antiviral capacity and immunity. Matrine may play an anti-PRRSV role by directly acting on ACAT1, ALB, HMOX1, HSPA8, HSP90AB1, PARP1 and STAT1.

## 1. Introduction

Porcine reproductive and respiratory syndrome virus (PRRSV) is an enveloped, single-stranded, positive-sense RNA virus that causes porcine reproductive and respiratory syndrome (PRRS) in clinical practice. It is characterized by reproductive disorders in pregnant sows, dyspnea and growth retardation in pigs of all ages. The infected animals showed immunosuppression and even secondary infection and co-infection [1]. Therefore, it seriously endangers the development of the pig industry. In addition to HP-PRRSV, several variants have been co-prevalent [2,3], which brings great difficulties to the prevention and control of PRRS. At present, the PRRSV vaccine cannot provide complete immune protection, and the incomplete attenuation procedure involved in the production of improved live vaccines may lead to the enhanced virulence of PRRSV [4,5]. Therefore, the current situation of PRRS prevention and control is still grim.

Traditional Chinese medicine not only has antiviral effects, but can also enhance the body’s immunity, showing its unique advantages in the clinical prevention and treatment of PRRS [6]. We have been committed to the study of new antiviral veterinary drugs for a long time. We screened and found that matrine has an anti-PRRSV effect [7,8,9], and revealed its mechanism from the perspective of the PRRSV infection process and autoimmunity. Matrine can inhibit the expression of N protein [7] and inhibit the production of IL-1β by inhibiting the MyD88/NF-κB signaling pathway and the activation of NLRP3 inflammasome [10]; therefore, it exerts anti-inflammatory and antiviral activity.

However, the process of the natural compound, from its discovery to new drug approval and application, is time-consuming and expensive. One of the reasons is that it is difficult to understand its mechanism and potential side effects. Small molecules usually achieve their functions and exert pharmacological effects by interacting with protein targets. The identification of protein targets will help to clarify the mechanism of action and evaluate potential side effects. The increasingly developed multi-omics techniques, especially activity-based protein profiling (ABBP) [11], have recently been increasingly used for the target recognition of drugs and other bioactive small molecules. In this study, the anti-PRRSV activity and anti-inflammatory activity of two biotin-labeled matrine probes were evaluated in Marc-145 cells. The anti-PRRSV targets of matrine in Marc-145 cells were identified via ABPP, and combined with the predicted targets of network pharmacology. Then, GO analysis and KEGG analysis were used to explore the possible antiviral mechanism of matrine.

## 2. Results

### 2.1. Cytotoxic Effects of Compounds on Marc-145 Cells

**Figure 1:**
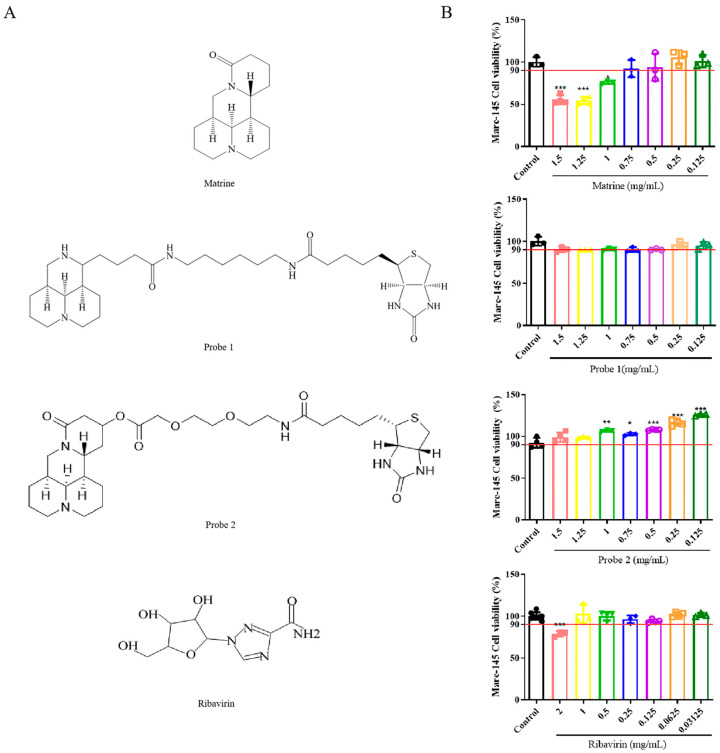
Cytotoxicity of compounds on Marc-145 cells. (**A**) Chemical structures of all compounds. (**B**) MTT assay was used to detect the activity of Marc-145 cells in each treatment group. The red line indicates 90% of cell viability. The data are represented as the mean ± SD from at least three samples and three experiments that showed similar results using one-way ANOVA. * represents a significant difference compared with the cell control group. * *p* < 0.05; ** *p* < 0.01; *** *p* < 0.001.

The chemical structures of the compounds are shown in Figure 1A. Biotin-labeled matrine probe 1 and 2 were synthesized via hydrolysis ring-opening and non-ring-opening, respectively. The results of MNTC determined via MTT assay (Figure 1B) showed that matrine had no cytotoxic effect on Marc-145 cells when the concentration was less than or equal to 0.75 mg/mL. Probe 1 and probe 2 had no cytotoxicity on Marc-145 cells in the tested concentration range, and the cell viability was greater than 90%. When the concentration of ribavirin was less than or equal to 1 mg/mL, the cell viability was greater than 90%. Therefore, the MNTC of each compound on Marc-145 cells was as follows: matrine 0.75 mg/mL; probe 1 1.5 mg/mL; probe 2 1.5 mg/mL; ribavirin 1 mg/mL. MNTC was selected for subsequent experiments.

### 2.2. Probes Retained Anti-PRRSV Activity

**Figure 2:**
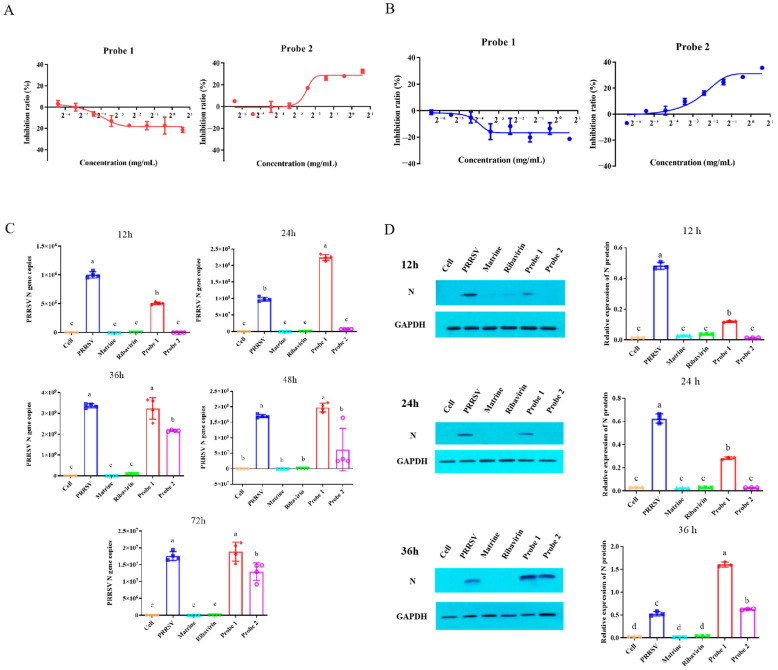
Identification of probe anti-PRRSV activity. (**A**) PRRSV was infected and then treated with probe. The inhibition rate of probe 1 and probe 2 on PRRSV-infected Marc-145 cells was detected via MTT assay. The curve showed that the inhibition effect of probe on PRRSV was dose-dependent. *n* = 3. (**B**) PRRSV was co-treated with probe, and the inhibition rate of probe 1 and probe 2 on PRRSV-infected Marc-145 cells was detected via MTT assay. The curve showed that the inhibition effect of probe on PRRSV was dose-dependent. *n* = 3. (**C**) qPCR was used to detect the expression of PRRSV N gene at 12 h, 24 h, 36 h, 48 h and 72 h after PRRSV and probe co-treatment. The data are represented as the mean ± SD from at least three samples and three experiments that showed similar results via one-way ANOVA. Different letters (a–c) indicate significant differences between groups, *p* < 0.05. (**D**) Western blot was used to detect the expression of PRRSV N protein at 12 h, 24 h and 36 h after PRRSV and probe co-treatment. The data are represented as the mean ± SD from at least three samples and three experiments that showed similar results via one-way ANOVA. Different letters (a–d) indicate significant differences between groups, *p* < 0.05.

The inhibition rate of probe 1 and probe 2 on PRRSV-infected Marc-145 cells was determined via MTT assay. The highest inhibition rate of PRRSV was 3.3% and 32.3 for probe 1 and probe 2, respectively, when the probe treatment started before the PRRSV infection (Figure 2A). In the case of PRRSV infection and probe treatment simultaneously (Figure 2B), the inhibition rates of probe 1 and probe 2 became −1.3% and 35.7%, respectively.

The effect of probe 1 and probe 2 on PRRSV N gene expression was detected via qPCR (Figure 2C). The results showed that the expression of PRRSV N gene in 12h PRRSV-infected Marc-145 cells in probe 1 and probe 2 groups decreased significantly (*p* < 0.05), compared with that of the PRRSV group. Compared with the probe 1 group, the expression of the PRRSV N gene in the probe 2 group was significantly decreased (*p* < 0.05). Compared with the PRRSV group, the expression of the PRRSV N gene in 24–72 h PRRSV-infected Marc-145 cells in the probe 2 group decreased significantly (*p* < 0.05). The results showed that both probe 1 and probe 2 had an anti-PRRSV effect on 12 h PRRSV-infected Marc-145 cells. For 24–72 h PRRSV-infected cells, probe 1 had no anti-PRRSV effect, and probe 2 had a significant anti-PRRSV effect.

The effects of probe 1 and probe 2 on PRRSV N protein expression were detected via Western blot (Figure 2D). The results showed that the expression of PRRSV N protein in 12–24 h PRRSV-infected Marc-145 cells in probe 1 and probe 2 groups decreased significantly (*p* < 0.05) compared with that of the PRRSV group. Compared with the probe 1 group, the expression of the PRRSV N protein in the probe 2 group was significantly decreased (*p* < 0.05). Compared with the PRRSV group, the expression of the PRRSV N protein in 36 h PRRSV-infected Marc-145 cells in the probe 1 and probe 2 groups was significantly increased (*p* < 0.05), and the expression of the PRRSV N protein in the probe 2 group was significantly lower than that in the probe 1 group (*p* < 0.05). The results showed that for PRRSV-infected Marc-145 cells for 12–24 h, probe 1 and probe 2 had an anti-PRRSV effect, and the anti-PRRSV effect of probe 2 was stronger than that of probe 1. When Marc-145 cells were infected with PRRSV for 36 h, probe 1 and probe 2 had no anti-PRRSV effect. In summary, both probe 1 and probe 2 partially retained the anti-PRRSV activity of matrine, and the effect of probe 2 was better than that of probe 1.

### 2.3. Probes Retained Anti-Inflammatory Activity

**Figure 3:**
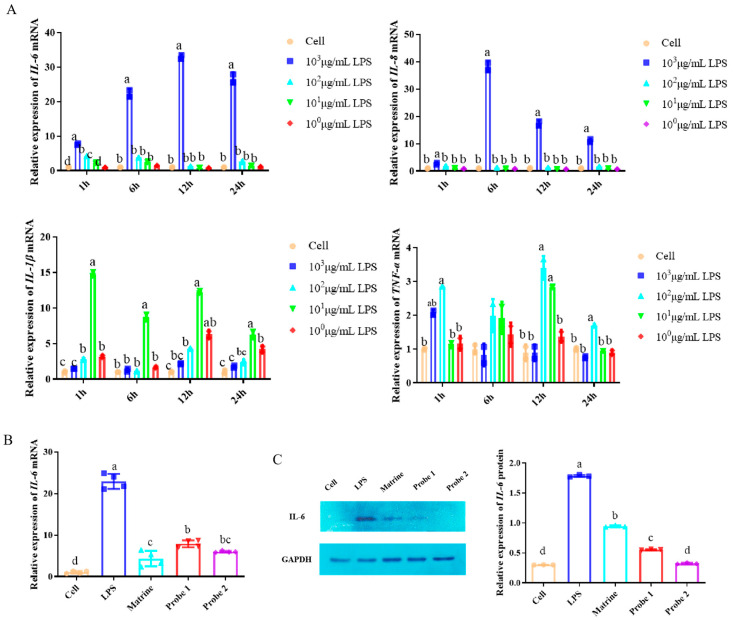
Identification of anti-inflammatory activity of probes. (**A**) qPCR was used to detect the expression of IL-6, IL-8, IL-1β and TNF-α mRNA at different time and LPS concentration, and to optimize the modeling conditions of cell inflammation model. The data are presented as the mean ± SD from at least three samples and three experiments that showed similar results via one-way ANOVA. Different letters (a–d) indicate significant differences between groups, *p* < 0.05. (**B**) The effect of probes on LPS-induced IL-6 mRNA expression was detected via qPCR. The data are represented as the mean ± SD from at least three samples and three experiments that showed similar results via one-way ANOVA. Different letters (a–d) indicate significant differences between groups, *p* < 0.05. (**C**) Western blot was used to detect the effect of probes on LPS-induced IL-6 protein expression. The data are represented as the mean ± SD from at least three samples and three experiments that showed similar results via one-way ANOVA. Different letters (a–d) indicate significant differences between groups, *p* < 0.05.

The relative mRNA expression levels of IL-6, IL-8, IL-1β and TNF-α in Marc-145 cells treated with LPS for 1 h, 6 h, 12 h and 24 h were detected via qPCR (Figure 3A), and the optimal modeling conditions of LPS on Marc-145 cells were screened. The results showed that the relative expression of IL-1β and TNF-α mRNA in Marc-145 cells treated with LPS for 1–24 h was at a low level. The relative expression of IL-8 mRNA was higher at 6 h and lower at 12–24 h. The relative expression of IL-6 mRNA was at a high level at 6–24 h, indicating a good pro-inflammatory effect. The relative expression was the highest at 12 h and 10^3^ μg/mL. Therefore, it was determined that treatment with 10^3^ μg/mL LPS for 12 h was the best condition for establishing an inflammatory model on Marc-145 cells.

The relative expression levels of IL-6 mRNA and IL-6 protein in Marc-145 cells treated with LPS and probe for 12 h were detected via qPCR and Western blot, respectively. The results of qPCR (Figure 3B) showed that the relative expression of IL-6 mRNA in LPS group was significantly higher than that in cell control group (*p* < 0.05), indicating that the inflammatory model was successfully established. Compared with the LPS group, the relative expression of IL-6 mRNA in the probe 1 and probe 2 groups was significantly decreased (*p* < 0.05). Western blot results (Figure 3C) showed that the relative expression of IL-6 protein in the LPS group was significantly higher than that in the cell control group (*p* < 0.05). Compared with the LPS group, the relative expression of IL-6 protein in the probe 1 and probe 2 groups was significantly decreased (*p* < 0.05). The results showed that probe 1 and probe 2 had significant anti-inflammatory effects.

### 2.4. Identification of Potential Targets of Matrine

**Figure 4:**
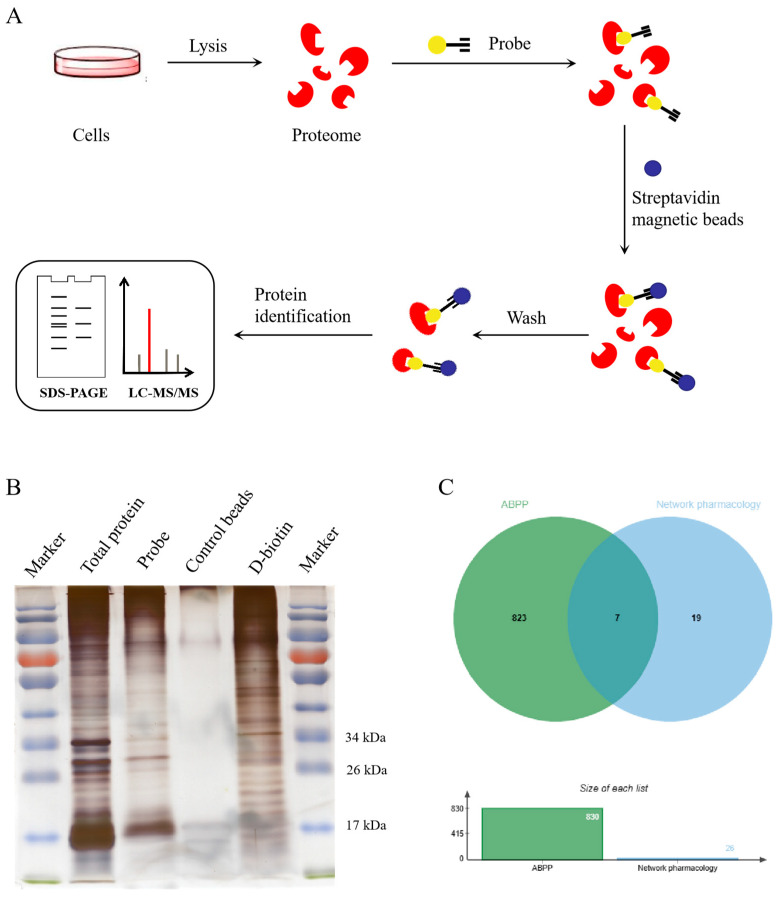
Molecular target fishing experiment. (**A**) ABPP target fishing experiment flow chart. The biotin-labeled matrine probe was co-incubated with the cell lysate. After incubation with streptavidin magnetic beads, the matrine target proteins were enriched on the streptavidin magnetic beads. (**B**) SDS-PAGE-silver nitrate staining was used to identify the target fishing results. The probe group showed strong protein bands around 17 kDa and 26–34 kDa, indicating potential targets for matrine. (**C**) The intersection of ABPP identification results and network pharmacology prediction targets.

Based on the results of anti-PRRSV and anti-inflammatory effects of the probes, probe 2 was selected for target fishing. The experimental flow chart was shown in Figure 4A. After the target protein binds to the biotin-labeled matrine probe, the streptavidin magnetic beads adsorb the biotin-labeled matrine and also adsorb the target protein to achieve the effect of enriching the target protein. The target protein SDS-PAGE electrophoresis gel was stained with silver nitrate (Figure 4B). The probe group showed strong protein bands around 17 kDa and 26–34 kDa, indicating potential targets for matrine. Among the total of 830 proteins identified via LC-MS/MS analysis, 26 of them were intersected with matrine anti-PRRSV targets screened via network pharmacology [12], and seven candidate targets were screened (Figure 4C): ACAT1, ALB, HMOX1, HSPA8, HSP90AB1, PARP1, STAT1. Target information was shown in Table 1.

### 2.5. Functional Analysis of Potential Target Proteins of Matrine

**Figure 5:**
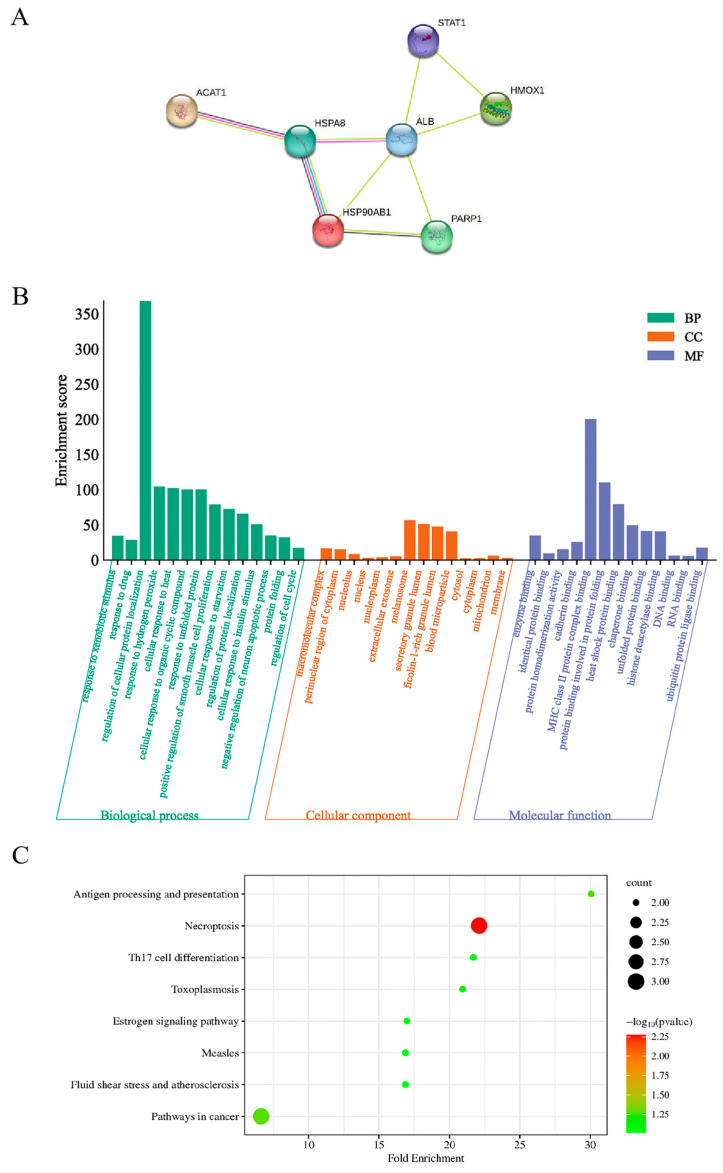
Bioinformatics analysis of potential targets of matrine. (**A**) Protein–protein interaction network. (**B**) GO function analysis. (**C**) KEGG pathway enrichment analysis.

The seven selected candidate proteins were input into the String database to obtain the protein–protein interaction network (Figure 5A). The number of nodes was seven, the number of edges was nine, the expected number of edges was four, the average node degree value was 2.57, the average local clustering coefficient was 0.757, and the PPI enrichment P value was 0.0213. This means that there may be more interactions between these seven proteins, which are synergistic or interlinked in some biological functions.

Furthermore, GO function analysis was performed on the seven candidate proteins (Figure 5B). It was found that the target protein is mainly involved in the following biological processes such as response to xenobiotic stimulus, response to drug, regulation of cellular protein localization, protein folding and regulation of cell cycle. The macromolecular complex, perinuclear region of cytoplasm, nucleolus and other cellular components were enriched. The molecular functions of these targets were mainly related to enzyme binding, identical protein binding, protein binding involved in protein folding, etc. KEGG analysis was mainly enriched in necroptosis and pathways in cancer (Figure 5C).

## 3. Discussion

Although our understanding of PRRS has greatly improved, there is still an urgent need for effective treatment and prevention strategies. As a potential preventive and therapeutic drug [6], small molecules of traditional Chinese medicine need to be explored for a clearer mechanism of action to promote research and development. The revelation of drug targets is of great guiding significance for elucidating the mechanism of drug action. With the progress of research in recent years, the ABPP method is becoming increasingly popular as a powerful tool for target mining [13]. Based on the ABPP method, Celastrol’s targets for improving liver fibrosis were identified as PRSXs and HO-1 [14], and Capsaicin’s targets for inhibiting sepsis were PKM2-LDHA [15]. Through the combination of ABPP and CETSA, eight proteins were identified as antimalarial targets of chloroquine [16]. In this study, we used ABPP to screen the targets of matrine against PRRSV-infected Marc-145 cells. Generally, it includes probe synthesis, target fishing and protein identification [13].

In this study, two biotin-labeled matrine probes were constructed via structural modification, which were probe 1 with an amide ring opening, and probe 2 without a ring opening. The activity of each probe was verified via cell experiments. Both probe 1 and probe 2 partially retained the anti-PRRSV activity and anti-inflammatory activity of matrine, but the antiviral effect gradually weakened with the prolongation of action time. Probe 2 showed better anti-PRRSV activity than probe 1, suggesting that the amide ring may be an essential part of the matrine structure for its anti-PRRSV effect. After the ring-opening treatment of matrine, its pharmacological effects were affected. The compound showed good anti-TMV activity in vitro and in vivo because the open amide ring of matrine reduced the C11 side chain to butyl and introduced sulfonyl, acyl, alkyl or carbamoyl into nitrogen [17]. When the ring of the matrine was opened, different groups were added to the end of its N12 and C11 side chains to synthesize a series of new derivatives, which showed better anti-CVB3 activity [18]. The introduction of the benzyl group at the N-position showed good anti-HBV activity [19]. However, in the case of the non-ring opening of matrine, the introduction of different groups at the C13 position has also been reported to enhance its activity. The matrine derivative ZS17, which was designed and synthesized under the condition of retaining the amide ring, had a more effective anti-tumor effect than matrine itself by activating the ROS-JNK-P53 signaling pathway [20]. The derivatives showed stronger insecticidal and fungicidal activities than matrine when the halogenated pyrazole groups were introduced on matrine C13 [21]. Finding the active part of the compounds is conducive to the study of its mechanism and the development and utilization of new veterinary drugs. This experiment proved that the matrine amide ring is a favorable target for the development of anti-PRRSV matrine derivatives.

Based on the results of the probes’ anti-PRRSV and anti-inflammatory activity tests, probe 2 was selected for target fishing, and probe 2 was co-incubated with Marc-145 cell lysate to achieve the binding of matrine to the targets. Biotin specifically binds to streptavidin magnetic beads to achieve the effect of enriching target proteins. SDS-PAGE-silver nitrate staining showed that the probe group had specific bands and successfully enriched specific proteins. Through mass spectrometry identification, combined with network pharmacology results, seven candidate targets were determined, which were ACAT1, ALB, HMOX1, HSPA8, HSP90AB1, PARP1 and STAT1.

All these potential targets of matrine are related to viral infection and immunity. ACAT1 is a cholesterol regulatory enzyme, and many viruses rely on cholesterol for effective infection. The presence of cholesterol is an essential part of PRRSV infection. The consumption of cholesterol can significantly inhibit the invasion, adhesion and release of PRRSV [22]. PRRSV NSP4 up-regulates cellular cholesterol to inhibit the production of type I interferon and evade host antiviral innate immunity [23]. The decrease in ALB content will reduce the synthesis of immunoglobulin, and the immune function of the body is at a low level, which will increase the probability of the body being infected by external pathogens. Clinical data have shown that ALB levels are associated with the severity of COVID-19 patients, and patients with higher ALB levels (>35.8 g/L) show a better prognosis [24]. Matrine can up-regulate the expression of ALB in the liver injury model in a dose-dependent manner [25]. HMOX1 is a rate-limiting enzyme in heme catabolism and has shown significant antiviral effects [26,27,28]. The overexpression of HMOX1 attenuates PRRSV replication in Marc-45 cells and PAM cells [29]. Liu L used proteomics technology to identify and analyze the interaction between PARP1 and PRRSV N protein, which can be considered as a positive viral factor. Inhibition of PARP-1 significantly reduced the production of viral genomic and subgenomic RNAs, indicating that PARP-1 may be recruited by the N protein and facilitate viral RNA replication and transcription [30]. STAT1 mediates cell response to interferon, and IFN signaling is an important mechanism for viruses to evade host innate immune response. PRRSV NSP1β interferes with IFN-α signaling by blocking the nuclear translocation of STAT1 [31], and IFN signaling has a physiological correlation with porcine PRRSV infection. However, whether these matrine-targeted proteins exert anti-PRRSV function remains to be further determined.

It is worth noting that two of the seven proteins screened in this study belong to the heat shock protein family. HSP90AB1 and HSPA8 are involved in multiple GO entries and KEGG signaling pathways, and are strongly associated with PRRSV infection. HSPA8 is a member of the heat shock protein family and has been shown to contribute to a variety of viral infections [32] such as DENV [33], JEV [34], and EBOV [35]. Studies have shown that HSPA8 interacts with PRRSV GP4 through its PB domain and participates in the attachment and internalization of PRRSV [36]. HSP90AB1, a member of the HSP90 family, is involved in the immune and inflammatory responses to COVID-19 [37], ASFV [38], and PDCoV [39]. It is an attractive antiviral target. Studies have shown that HSP90 expression increased in PRRSV-induced porcine lung injury model [40], and HSP90 inhibitors have strong anti-PRRSV activity through cell experiment [41]. Interestingly, matrine showed a high affinity with HSP90, and matrine could restore axonal growth and function in mice with spinal cord injury by activating HSP90 [42]. On the other hand, new progress has been made in the design of matrine derivatives based on the activity of HSP90 inhibitors [43]. Our latest study showed that infection with PRRSV induced a significant increase in the expression of HSPA8 and HSP90AB1, while matrine treatment could significantly reverse it, and the number of PRRSV viruses also decreased [12]. The results further support that HSP90AB1 and HSPA8 are potential targets of matrine against PRRSV; however, whether they directly interact with matrine needs further experimental verification.

## 4. Materials and Methods

### 4.1. Compounds, Cell and Virus

Matrine (CAS: 519-02-8) was purchased from the National Institutes for Food and Drug Control, with a purity of 98.7%. Ribavirin (CAS: 36791-04-5) was purchased from Solarbio with a purity of 99%. Probe 1 was synthesized by Shanghai NF Biotechnology Co., Ltd. (Shanghai, China). Probe 2 was synthesized and presented by Associate Professor Chunli Wu from Zhengzhou University. Marc-145 cells were purchased from the China Institute of Veterinary Drugs Control (Beijing, China). The cell culture medium was DMEM medium containing 10% FBS, and the cell maintenance medium was DMEM medium containing 2% FBS. PRRSV was extracted from the diseased material and proliferated. The TCID_50_ in Marc-145 cells was 10^−7.79^/mL using the Reed–Muench method [44].

### 4.2. Cytotoxicity Assay

The compounds were serially diluted into 7 concentrations with cell maintenance medium and added into the 96-well plate of Marc-145 cells, which was cultured in a 5% CO_2_ cell incubator at 37 °C. To the control group, only the cell maintenance medium was added. After 72 h of incubation, the medium containing compounds was discarded; then, 20 μL MTT was added to each well and these were further incubated for 4 h at 37 °C. The MTT was then removed and 150 μL DMSO was added to dissolve the formazan crystals. The optical density was measured using a microplate reader at 490 nm wavelength. The maximum non-cytotoxic concentration (MNTC) of a compound with 90% cell viability was determined.

### 4.3. The Inhibition Rate of Probes on PRRSV-Infected Marc-145 Cells

PRRSV and probe were treated in two ways. PRRSV (100 TCID_50_/mL) was added to the 96-well plate of Marc-145 cells. After viral adsorption for 2 h, the supernatant was discarded and the probe was added at 8 concentrations diluted with cell maintenance medium. Alternatively, the gradient diluted probe and PRRSV were simultaneously added to Marc-145 cells. To the control group, only the cell maintenance medium was added, and to the virus group only PRRSV was added. After 72 h of culture, the OD value of each well was detected via MTT assay, and the inhibition rate (%I) of the probe on PRRSV-infected Marc-145 cells was calculated using the following formula [45]: %I = (OD_test_ − OD_virus_)/(OD_control_ − OD_virus_) × 100%.

### 4.4. Detection of PRRSV N Gene/Protein Expression

The compound and PRRSV were added to the 6-well plate of Marc-145 cells at the same time; the final concentration of the compound was MNTC, and the final concentration of PRRSV was 100 TCID_50_/mL. To the cell group, only the cell maintenance medium was added, and to the virus group, only PRRSV was added. After 12 h, 24 h, 36 h, 48 h and 72 h, the cells were collected for the subsequent extraction of total RNA and total protein. The expression of PRRSV N gene was detected via qPCR. The expression of PRRSV N protein was detected via Western blot.

### 4.5. Establishment of Inflammatory Cell Model

The 10^3^ μg/mL LPS was serially diluted with cell maintenance medium for 4 concentration gradients (10-fold ratio) and added to Marc-145 cell 6-well plates. To the cell group, only the cell maintenance medium was added. The cells were collected after 1 h, 6 h, 12 h and 24 h of culture, and the total RNA was extracted. The expression of IL-6, IL-8, IL-1β and TNF-α mRNA was detected via qPCR.

### 4.6. Determination of IL-6 mRNA/Protein Expression

LPS and compound were simultaneously added to the 6-well plate of Marc-145 cells; the final concentration of the compound was MNTC, and the final concentration of LPS was 10^3^ μg/mL. To the cell group, only the cell maintenance medium was added, and to the LPS group, only LPS was added. After 12 h of culture, the cells were collected, and the total RNA was extracted. The expression of IL-6 mRNA was detected via qPCR. β-actin was set as the housekeeping gene, and the relative expression of IL-6 mRNA was calculated using the 2^−ΔΔCt^ method. The expression of IL-6 protein was detected via Western blot.

### 4.7. qPCR

The total RNA of Marc-145 cells was extracted using TRIzol reagent (TaKaRa, Beijing, China), and the RNA concentration was detected using an ND-1000 spectrophotometer (NanoDrop Technologies, Wilmington, DE, USA). The cDNA was synthesized using a Prime Script™ RT Reagent kit with gDNA eraser (TaKaRa, Beijing, China). SYBR Green qPCR Master Mix (Bimake, Houston, TX, USA) fluorescence quantitative reagent and ABI 7500 Real-Time PCR system were used for PCR tests. The expression of the PRRSV N gene was detected via absolute quantitative PCR, and the standard curve was constructed with 10 times serial dilutions of PRRSV N gene recombinant plasmid. The relative expression levels of inflammatory factors IL-6, IL-8, IL-1β and TNF-α mRNA were calculated using the 2^−ΔΔCt^ method. The primers used for qPCR are presented in Table 2.

### 4.8. Western Blot

The total protein of Marc-145 cells was extracted using RIPA lysate (Solarbio, Beijing, China), and the protein concentration was determined using the BCA protein assay kit (Beyotime, Shanghai, China). The same amount of protein was separated with an SDS-PAGE gel using PageRuler Prestained Protein Ladder (Thermo, Waltham, MA, USA) and then transferred to the PVDF membrane. The PVDF membrane was then cut into strips according to the predicted molecular weight of the target protein. The PVDF membrane was blocked in TBST containing 5% skimmed milk powder at room temperature for 2 h, and then incubated with GAPDH antibody (1:5000, Proteintech, Wuhan, China), PRRSV N antibody (1:500), and IL-6 antibody (1:500, Thermo, Waltham, MA, USA) at 4 °C overnight, respectively. After washing with TBST 3 times, it was incubated with HRP-Goat Anti-Mouse IgG (1:20,000, CWBIO, Jiangsu, China) secondary antibody at room temperature for 1 h, and then washed with TBST 3 times. The immunoreactive proteins were visualized using an eECL chemiluminescent detection kit (Boster, Wuhan, China) and detected using X-ray films. Image J 1.42q software (National Institutes of Health, Bethesda, MD, USA) was used for gray value analysis of the target protein band.

### 4.9. Target Fishing

The total protein of Marc-145 cells was extracted with RIPA lysate and incubated with biotin-labeled matrine probe, which retained pharmacological activity at 4 °C for 6 h to enrich the proteins interacting with matrine. Streptavidin magnetic beads (Purimag, Xiamen, China) were added and incubated overnight at 4 °C. The affinity of biotin and streptavidin was used to form a compound matrix, and the magnetic beads were then collected through a magnetic frame. The beads were washed 3 times with PBS and the unbound proteins were eluted. To the control beads group, only streptavidin magnetic beads was added, and in the D-biotin group, the probe was replaced with D-biotin. Some of the collected magnetic beads were used for SDS-PAGE-silver nitrate staining, and some were used for LC-MS/MS protein identification.

### 4.10. SDS-PAGE-Silver Nitrate Staining

The beads combined with targeted matrine protein were added to the protein loading buffer and denatured at 95 °C for 10 min so that the proteins were fully denatured and separated from the magnetic beads for SDS-PAGE gel electrophoresis. The gel was then washed, fixed, eluted, washed, sensitized, silver stained, washed, developed and terminated to identify the target group of matrine according to the instructions of the silver nitrate staining kit (CWBIO, Taizhou, China).

### 4.11. LC-MS/MS

The beads combined with targeted matrine protein were subjected to trypsin digestion, C18 desalination, mass spectrometry and data retrieval. The target protein was analyzed using liquid chromatography–mass spectrometry (LC-MS/MS). The name of the potential binding target protein was determined using the Uniprot database sequence alignment method.

### 4.12. Conjoint Analysis

The results of LC-MS/MS mass spectrometry and the results of network pharmacology analysis, which we have reported previously [12], were intersected to screen out the candidate proteins for matrine to exert antiviral effects. The protein–protein interaction network (PPI) was drawn using the String database (https://string-db.org/ (accessed on 10 February 2023)) with default values [46]. GO analysis and KEGG pathway analysis of candidate proteins were performed using the DAVID database (https://david.ncifcrf.gov/ (accessed on 10 February 2023)) [47].

### 4.13. Statistical Analysis

All the data were expressed as mean ± SD. Data analysis was performed using one-way ANOVA in GraphPad Prism 8 software (GraphPad software, San Diego, CA, USA). * *p* < 0.05; ** *p* < 0.01; *** *p* < 0.001. The different letters such as a, b, c and d indicated that the values were significantly different, *p* < 0.05.

## 5. Conclusions

Based on the ABPP method, we explored the protein targets of matrine against PRRSV infection in Marc-145 cells, and identified ACAT1, ALB, HMOX1, HSPA8, HSP90AB1, PARP1 and STAT1 as potential candidates, which are related to antiviral and immune properties. The pharmacological activity of matrine against PRRSV involves an extremely complex mechanism. Further experiments are still needed to explore its direct targets and cascade regulation of signaling pathways. The results of this study provide a new insight of the mechanism of matrine against PRRSV. At the same time, this study provides ideas for screening or designing antiviral drugs for targets from a new perspective of “target-driven” pharmacological research of traditional Chinese medicine.

## Figures and Tables

**Table 1:** Potential protein information.

No.	Gene	Description
1	ACAT1	Acetyl-CoA acetyltransferase
2	ALB	Albumin
3	HMOX1	Heme oxygenase 1
4	HSPA8	Heat shock cognate 71 kDa protein
5	HSP90AB1	Heat shock protein HSP 90-beta
6	PARP1	Poly [ADP-ribose] polymerase 1
7	STAT1	Signal transducer and activator of transcription 1-alpha/beta

**Table 2:** Primer sequences.

Genes	Primer Sequences
PRRSV N	F: AGAAGCCCCATTTCCCTCTA R: CGGATCAGACGCACAGTATG
β-actin	F: TGGTGGGCATGGGTCAGAAGG R: ATGGGGTACTTCAGGGTGAGGATG
IL-6	F: GGTGTTGCCTGCTGCCTTCC R: TGAGATGCCGTCGAGGATGTACC
IL-8	F: AGCTGGCGGTGGCTCTCTTG R: TGGGGTGGATAGGTTTGGAGTACG
IL-1β	F: ACCTATCTTCCTCCACACAAGC R: TCTGCTTGAGAGGTGCTGATG
TNF-α	F: TGTGTCTGCTGCACTTTGGAGTG R: TTGAGGGTTTGCTACAACATGG

## Data Availability

The data generated/analyzed during the current study are available from the corresponding author upon request.
